# Promoting adolescents' pro‐environmental behavior: A motive‐alignment approach

**DOI:** 10.1111/jora.13044

**Published:** 2024-12-10

**Authors:** Judith van de Wetering, Stathis Grapsas, Astrid Poorthuis, Sander Thomaes

**Affiliations:** ^1^ Department of Psychology Utrecht University Utrecht The Netherlands; ^2^ Department of Education and Pedagogy Utrecht University Utrecht The Netherlands

**Keywords:** adolescence, autonomy, motive‐alignment, pro‐environmental behavior

## Abstract

Most adolescents are concerned about climate change. What helps them to act on their concerns? This preregistered randomized experiment tested whether adolescents' pro‐environmental behavior can be promoted by framing the behavior as compatible with their autonomy motive. Dutch adolescents (*N* = 319, ages 12–17, 57.7% girls, predominantly indicating “Dutch” or “bicultural” identities) viewed a campaign‐style video that explained the causes of climate change (all conditions), and additionally framed pro‐environmental behavior as a personal choice (volition‐alignment), opportunity to rebel (rebellion‐alignment), or mandatory (misalignment). Rebellion‐alignment increased pro‐environmental behavior intentions and petitioning behavior; misalignment decreased pro‐environmental donating behavior. Effect sizes were small to medium. These findings provide proof of concept that motive alignment can be effective in promoting adolescents' pro‐environmental engagement.

## INTRODUCTION

Adolescents are motivated to make meaningful contributions to society (Crone & Fuligni, 2019; Fuligni, 2019). One of the biggest threats to the world today is climate change. Over the past years, young activists around the world have initiated street protests, engaged in online activism, and filed lawsuits to take a stand against what they perceive as climate inaction of world leaders and big industries (Bandura & Cherry, 2020; de Moor, Uba, et al., 2020; Wahlström et al., 2020). Most adolescents worry about climate change, sometimes excessively (Corner et al., 2015; Sanson et al., 2019). A public opinion survey found that approximately two out of three adolescents around the world consider climate change a ‘global emergency’ (United Nations Development Programme [UNDP], 2021).

One might perhaps expect, then, that adolescents show high rates of pro‐environmental behavior (i.e., any behavior that benefits the natural environment, or harms it as little as possible; Steg & Vlek, 2009) in their everyday lives. Research, however, suggests otherwise. Despite their concern about climate change, adolescents are less likely than both younger children and adults to engage in pro‐environmental behavior (Otto & Kaiser, 2014; UNDP, 2021). The present experiment provides a proof‐of‐concept test of a novel, theory‐driven approach to promote adolescents' pro‐environmental behavior. The basic premise of our approach is that effective behavior change promotion should align pro‐environmental behavior with adolescents' core motives. We broadly conceptualize motives as the needs, goals, and desires that adolescents deeply care about in their daily lives (Dweck, 2017; Thomaes et al., 2023). In a preregistered randomized experiment, we tested whether adolescents can be effectively encouraged to engage in pro‐environmental behavior when such behavior is framed as an opportunity to satisfy their motive for autonomy—that is, when pro‐environmental behavior is motive‐aligned (Fuligni, 2019; Thomaes et al., 2023; Yeager et al., 2018).

### Traditional policies to promote Adolescents' pro‐environmental behavior

Traditional policies to promote adolescents' pro‐environmental behavior include school‐based educational programs and public campaigns. The theory of change that underlies many of these policies holds that adolescents' pro‐environmental engagement can be promoted by teaching them knowledge and awareness of environmental problems, as well as behavioral skills to help address these problems (Borg et al., 2012; Kollmuss & Agyeman, 2010; Steg & Vlek, 2009). This logic is based on decision‐making theories that propose that individuals' awareness of environmental issues and the norms that surround them, along with the behavioral skills to help mitigate these problems, are key drivers of behavior change (Ajzen, 1991; de Leeuw et al., 2015; Fishbein & Ajzen, 2010; Klöckner & Blöbaum, 2010). Indeed, research has shown that traditional efforts that use educational techniques can be effective at promoting climate awareness and knowledge, and, to a lesser extent, pro‐environmental behavior in young people (Stern et al., 2014; van de Wetering et al., 2022). We propose, though, that the effectiveness of these policies may be strengthened by considering lessons learned from the developmental science of adolescence (Thomaes et al., 2023; Yeager et al., 2018).

First, adolescents are typically driven to act upon concerns salient to them in the here and now—they show increased neurological and behavioral responses to immediate rewards, and are disinclined to weigh long‐term outcomes in the decisions they make, compared to both children and adults (Blakemore & Robbins, 2012; Chein et al., 2011; Crone & Dahl, 2012; O'Brien et al., 2011). Traditional policies, however, often emphasize long‐term horizons (e.g., by pointing out how climate change may have devastating impacts in a distant future, or how pro‐environmental behavior has benefits that unfold over an extended time period). This long‐term emphasis may resonate less with adolescents' here and now orientation, thus limiting policy effectiveness (Yeager et al., 2018).

Second, adolescents are strongly motivated to acquire autonomy, an important developmental task of this age period (Fuligni, 2019). The transition from childhood into adolescence is marked by rapid increases in the desire to act on one's own interests and values, the belief that one is ready to make independent choices, and the tendency to resist others' attempts to influence one's behavior (Rodríguez‐Meirinhos et al., 2020; Ruck et al., 1998; Soenens et al., 2007). Notably, the extent to which adolescents succeed in experiencing autonomy varies across contexts and depends on the extent to which these contexts afford autonomy (Kuhn et al., 2020). For example, in educational contexts, adolescents often report experiencing less autonomy than they would like (Modrek et al., 2021; Modrek & Sandoval, 2020). And indeed, traditional educational policies to promote pro‐environmental behavior are inherently somewhat prescriptive, to the extent that they tell adolescents how to behave in ways that benefit the environment. Such prescriptive approaches may trigger resistance, and even lead adolescents to reactively refrain from engaging in pro‐environmental behavior in an effort to remain autonomous (Thomaes et al., 2023; van Petegem et al., 2015; Yeager et al., 2018).

Given these challenges, how can we inform policies to promote adolescents' pro‐environmental behavior? Building on recent insights (Bryan et al., 2016, 2019; Galla et al., 2021; Yeager et al., 2018), we propose that policies will be more effective if they successfully communicate that pro‐environmental behavior can be immediately rewarding. Accordingly, we examine whether pro‐environmental policy effectiveness can be improved by not just informing adolescents, but also harnessing (i.e., aligning with and highlighting opportunities to satisfy) their autonomy motive.

### Autonomy motive‐alignment to promote Adolescents' pro‐environmental behavior

Conceptualizations of adolescent autonomy have somewhat divergently emphasized volitional and independent functioning. Conceptualizations that emphasize volitional functioning, primarily rooted in the Self‐Determination Theory literature, refer to autonomy as the degree to which one behaves with a sense of freedom and in accordance with personal beliefs and self‐views (Deci & Ryan, 1985, 2000; Soenens et al., 2007). From this perspective, the autonomy motive is present across the lifespan and gradually increases (Deci & Ryan, 2000; Dweck, 2017; Soenens et al., 2017). Adolescents are highly sensitive to whether their sense of volition is acknowledged or thwarted. For instance, they are more motivated to learn in school when they feel that teachers support their volitional functioning (Kuhn et al., 2020; Niemiec & Ryan, 2009) Also, they are more motivated to act pro‐environmentally when they perceive that their parents support their personal choices (Grønhøj & Thøgersen, 2017). Even subtle lexical cues that signal to adolescents that their autonomy is acknowledged can be impactful. For example, compared with volition‐thwarting messages (e.g., “you have to”), volition‐acknowledging messages (e.g., “you might”) can promote adolescents' self‐reported effort and graded performance in school‐based sports exercises or knowledge tests (Vansteenkiste et al., 2005; Vansteenkiste, Simons, Lens, et al., 2004; Vansteenkiste, Simons, Soenens, & Lens, 2004). Hence, in the current study, we tested the effects of both volition‐aligned (i.e., “it is your choice”) and prescriptive‐misaligned (“you have no choice”) messaging.

Other conceptualizations of autonomy, primarily rooted in the developmental science literature, emphasize independent functioning. From this perspective, the autonomy motive becomes especially salient during the developmental phase of adolescence—a time when youth experience an urge to make their own decisions, develop their identity, and seek their own place in society (Darling et al., 2008; Fuligni, 2019; Rodríguez‐Meirinhos et al., 2020; Van Petegem et al., 2015). One manifestation of this search for independence is (some) adolescents' tendency to distance themselves from or rebel against authorities, such as their parents (Darling et al., 2008; Soenens et al., 2007; Van Petegem et al., 2015). While the term rebellion may have some negative connotations, perhaps reviving obsolete notions of adolescence as a period of “storm and stress” (Arnett, 1999), adolescents' rebellious tendencies can motivate them to be engaged societal actors. Adolescents are increasingly capable of taking others' perspectives, and increasingly care about fairness and social justice (Bryan et al., 2016; Crone, 2013). These developmental changes can motivate them to speak out and take a stand for social justice, such as through protesting and campaigning. As such, adolescent rebellion has been conceptualized as a form of positive risk‐taking behavior, reflecting healthy development and civic growth (Amnå, 2012; Duell & Steinberg, 2019; Wray‐Lake et al., 2017, 2019).

Recent experiments have shown how adolescents' rebellious inclinations can be harnessed to promote behavior change (i.e., healthy eating; Bryan et al., 2016, 2019), or self‐regulation (i.e., limiting social media use; Galla et al., 2021). These experiments tested proof‐of‐concept interventions using so‐called motive‐alignment (or: values‐alignment; Bryan et al., 2016) techniques. These techniques align targeted behavior with adolescents' core motives, such as their autonomy motive: The experiments framed consuming healthy foods or reducing social media use as opportunities to take a stand against the deceptive and manipulative practices of big industries (Bryan et al., 2016, 2019; Galla et al., 2021). This message encouraged adolescents to reassert their independence from these industries by refusing to consume their products (Bryan et al., 2016, 2019), or to use their social media applications (Galla et al., 2021; Soenens et al., 2007). Thus, rather than prescribing behavior change, these interventions framed behavior change as compatible with adolescents' autonomy motive. Moreover, recent correlational work found evidence that adolescents who perceive pro‐environmental behavior as aligned with their motives are more likely to engage in such behavior (Grapsas et al., 2023). Hence, in the current experiment, we implemented autonomy‐alignment techniques, rooted in conceptualizations of autonomy both as volition and as rebellion, to promote adolescents' pro‐environmental behavior.

### The current experiment

Research suggests that adolescents' short‐term orientation and desire for autonomy can be leveraged to promote behavior change. In the current preregistered between‐subjects experiment conducted with Dutch adolescents (ages 12 to 17), we tested, for the first time, whether adolescents' pro‐environmental behavior can be promoted by aligning such behavior with their autonomy motive. We exposed adolescents to information that framed pro‐environmental behavior as either an opportunity to rebel against authority or a volitional expression of one's personal values. We hypothesized that, in the autonomy‐alignment (i.e., rebellion and volition) conditions, adolescents would be more likely to engage in pro‐environmental behavior, and report stronger pro‐environmental behavior intentions, compared to their counterparts in the control condition (who were exposed to educational information only). We also included an autonomy‐misalignment condition in which adolescents were exposed to information that framed pro‐environmental behavior as mandatory. We hypothesized that, in this condition, adolescents would be less likely to engage in pro‐environmental behavior, and report weaker pro‐environmental behavior intentions, compared to their counterparts in the control condition. We additionally explored whether the putative effects of the two autonomy‐alignment conditions (i.e., rebellion and volition) differed from each other.

## METHOD

### Ethics and Open Science statement

The study was approved by the Faculty of Social and Behavioral Sciences ethics review board at Utrecht University (protocol 20–641). We preregistered the study design, hypotheses, and analyses with the Open Science Framework (https://doi.org/10.17605/OSF.IO/Z93CF). The study materials and analysis code are accessible on OSF. The data that support the findings of this study are available on request from the corresponding author. The data are not publicly available due to privacy or ethical restrictions.

### Participants

Participants were 319 adolescents (57.7% girls), ages 12–17 (*M* = 14.63, *SD* = 1.28). They self‐identified mainly with Dutch (84.6%) or bicultural identities (11.9%). We recruited participants from five Dutch secondary schools. The Dutch secondary school system has three educational tracks, and participants were recruited from each track. Participants received a five‐euro reward after participation.

Data collection took place during the COVID‐19 pandemic, between April 2021 and February 2022. Due to restrictions at the time, a total of 60 participants took part in the experiment online, rather than in their classroom. These participants were evenly distributed across conditions, *χ* ^2^(3) = 0.54, *p* = .910. Due to the pandemic, we were unable to obtain our targeted sample size of 400 participants. We note, though, that our target was set to be very conservative (i.e., power > .90 to detect small to medium effects).

We sent out parental consent letters to a total of 768 adolescents who were eligible to participate. Of them, 433 (56.4%) received parental consent, 393 (51.2%) subsequently provided their own assent, and 319 (41.5%) took part in the experiment. Participant drop‐out was caused mostly by school absence due to pandemic disruptions.

### Procedure and measures

#### Baseline session

Up to 21 days (*M* = 6.43, *SD* = 4.95) prior to the experiment participants completed an online survey, assessing demographic information and variables not included in the present study.

#### Experimental session

The experiment proper was conducted in classrooms at participants' own schools. A trained experimenter instructed participants to individually complete the experiment on their laptops. The experimenter and the class teacher remained in class during the experiment, and were blind to condition assignment.

We randomly assigned participants to one of four conditions: a control condition (*N* = 79), rebellion‐alignment condition (*N* = 80), volition‐alignment condition (*N* = 81), or prescriptive misalignment condition (*N* = 79). In each experimental condition, participants individually watched a variation of a short (i.e., one‐ to two‐minute) video on deforestation and its contributions to climate change. We focused on deforestation because of its major environmental impacts, which are relatively easy to understand (DeFries et al., 2010; Hosonuma et al., 2012; Intergovernmental Panel on Climate Change [IPCC], 2019). Across conditions, the videos contained relevant images (i.e., melting icecaps, forests, a logging machine, a factory) and a voiceover narration by a young male. The voiceover explained that the earth is warming up due to CO_2_ emissions and that we can reduce global warming by protecting forests, which absorb CO_2_. The voiceover continued by explaining that tropical rainforest is cut down for the production of palm oil, which the food industry uses in their products. Then, the voiceover highlighted that environmental charities are committed to protecting the tropical rainforests and “tell big companies not to use palm oil for which forests have been cut down.”

For participants in the control condition, the video ended there. Thus, the video that they saw used a traditional educational approach that explains environmental problems and informs about actions to help counter them. For participants in the other three conditions, the video continued. In the rebellion‐alignment condition, the video appealed to autonomy conceptualized as reactance against authority figures and resistance to injustice (Bryan et al., 2016, 2019; Galla et al., 2021): it framed supporting pro‐environmental charities as a justified fight against exploitative companies (“These companies think it is more important to make profit than to protect the earth”, “If you want, you can rise against these unfair companies. How? For example, by supporting environmental organizations and actions that protect the tropical rainforest. Will you rise up?”). In the volition‐alignment condition, the video appealed to autonomy conceptualized as full willingness and psychological freedom (Deci & Ryan, 2000; Soenens et al., 2007): it framed supporting pro‐environmental charities as a personal choice (“If you want, you can support these organizations. Of course, doing so has to align with who you are and what you think. The choice is yours”). Finally, in the prescriptive misalignment condition, the video presented supporting pro‐environmental charities as an unavoidable duty (“Everyone must support these environmental organizations, including you. It does not matter who you are or what you think. Take action for the tropical rainforest. You have no other choice”).

##### Donation measure

Immediately after participants viewed the video, they were given the option to donate any amount of their five‐euro participation reward to one of four charities, including environmental (Rainforest Alliance, Environmental Defense Fund) and non‐environmental (International Rescue Committee, Amref Flying Doctors) ones. The charities were listed along with their logo and a short description of their mission. We included non‐environmental charities to be able to test the specificity of experimental effects for pro‐environmental behavior, rather than prosocial behavior more generally. We did not include very well‐known organizations (e.g., WWF or UNICEF) that could draw donations due to their relative familiarity (Smith & Schwarz, 2012). We coded pro‐environmental behavior dichotomously (i.e., donated to an environmental charity or not) because the amount of money that participants chose to donate was highly (right‐)skewed (skewness and kurtosis values were >2). Following university regulations, we could not donate on behalf of participants. We paid all participants the full five‐euro reward at the end of the study and explained them how to donate if they still wished to.

##### Petition measure

Next, participants were asked if they wanted to sign the ostensible #beclear petition, which asked a large food company to be transparent about the origins of the palm oil in its products, and about what they do to protect the rainforest. Signing the petition ostensibly entailed sending a digital postcard including the participant's first name and a short personal message directed to the company. We coded pro‐environmental behavior dichotomously (i.e., signed the petition or not). At the end of the study, we thoroughly debriefed participants. Regarding the petition, we informed participants that although the petition was not real, we would send a letter to the pertaining food company stating the number of adolescents who signed the petition, along with their personal messages.

##### Pro‐environmental behavior intentions

Next, we assessed intentions to engage in pro‐environmental behaviors to protect tropical rainforests using a three‐item scale tapping into environmental activism (“I plan to join actions to help protect the forests”), nonactivist behavior in the public sphere (“I am going to tell people around me how important it is that there are no trees being cut down in the tropical rainforest”), and private‐sphere behavior (“I want to try to eat or use less products that contain palm oil, because trees may have been cut down for those products”), as theoretically distinguished by Stern (2000; 1 = *totally disagree* to 5 = *totally agree*; *M* = 3.05, *SD* = 0.75, Cronbach's *α* = .68).

##### Robustness measures

We included one attention check item within the survey assessing pro‐environmental behavior intentions (i.e., “Select neutral for this item”); (Abbey & Meloy, 2017). We also asked participants at the end of the survey to indicate if they encountered any video or audio malfunction, and if they completed the study individually or together with others.

### Data analysis

#### Confirmatory analyses

As per our preregistration, we tested our hypotheses using linear and logistic regressions with planned contrasts, and regressed indices of pro‐environmental outcomes (pro‐environmental donations, petition‐signing, intentions) on experimental condition. First, to test the hypothesis that autonomy‐alignment would increase adolescents' pro‐environmental engagement, we contrasted both autonomy‐alignment conditions (i.e., rebellion, volition) with the control condition. Second, to test the hypothesis that autonomy‐misalignment would decrease adolescents' pro‐environmental engagement, we contrasted the autonomy‐misalignment condition with the control condition. We thus performed three tests per outcome variable (*α* < .05).

#### Robustness analyses

First, we tested whether the confirmatory analyses were robust (i.e., no change in direction and statistical significance of effects) to excluding participants who (a) encountered video malfunctions (visual, audio, or both), (b) indicated they did not complete the experiment individually, or (c) failed the attention check. Second, we tested whether the confirmatory analyses were robust in controlling for relevant demographic variables (i.e., those that correlated significantly with the outcome variable) and, although not preregistered, for whether participants completed the study in the classroom or online. Third, we corroborated our confirmatory tests with an alternative statistical approach—Bayesian informative hypothesis testing (Hoijtink et al., 2019)—to further explore the robustness of our findings (not preregistered).

#### Exploratory analyses

To explore whether effects differed between the two autonomy‐alignment conditions, we directly contrasted the rebellion‐alignment with the volition‐alignment condition. Additionally, we explored whether effects on the donation outcome measure would also hold for donating in general (including donations to both environmental and non‐environmental charities; not preregistered). Although we initially planned to explore whether the effects of the experimental manipulation on pro‐environmental outcomes were mediated by autonomy motive‐alignment, we dropped this analysis because we were unable to measure autonomy motive‐alignment reliably (Cronbach's *α* = .56; see Supplemental Material).

## RESULTS

### Preliminary analyses

Random assignment to conditions was successful: there were no condition differences in participants' age, *F* (3, 311) = 0.43, *p* = .732; gender, *χ* ^2^(3) = 4.29, *p* = .232; ethnic identity, *χ* ^2^(6) = 3.90, *p* = .690; educational level, *χ* ^2^ (6) = 0.81, *p* = .992; or online versus physical participation, *χ* ^2^(3) = 0.54, *p* = .910. We validated the fidelity of our experimental manipulations in an independent sample (see Supplemental Material). We found no impactful outliers (i.e., values both >3 *SD* from the mean and Cook's distance >1, as per the preregistration).

Table 1 presents descriptives. All pro‐environmental outcome measures correlated significantly (*r*s .19–.32, *p* < .01). On average, participants rated their behavioral intentions around the midpoint of the scale (*M* = 3.05, *SD* = 0.75). A relatively small proportion of participants decided to donate to environmental charity (12.6%) or sign the petition (16.3%).

**TABLE 1:** Descriptives of Study Variables.

Variable	*f* (%)	*M* (*SD*)
Pro‐environmental donating^a^	44 (13.8)	3.69 (1.64)^b^
Rebellion‐alignment	14 (17.5)	4.10 (1.59)^b^
Volition‐alignment	11 (13.6)	3.44 (1.85)^b^
Prescriptive misalignment	5 (6.3)	4.20 (1.79)^b^
Control	14 (17.7)	3.32 (1.51)^b^
Pro‐environmental petitioning	57 (17.9)	
Rebellion‐alignment	20 (25.0)	
Volition‐alignment	16 (19.8)	
Prescriptive misalignment	11 (13.9)	
Control	10 (12.7)	
Pro‐environmental behavior intentions		3.05 (0.75)
Rebellion‐alignment		3.17 (0.68)
Volition‐alignment		3.04 (0.74)
Prescriptive misalignment		3.05 (0.78)
Control		2.92 (0.77)

*Note*: Rebellion‐alignment *N* = 80, Volition‐alignment *N* = 81, Prescriptive misalignment *N* = 79, Control *N* = 79.

^a^ Includes donations to environmental charity only.

^b^ Based on the subset of participants who donated to environmental charity.

We explored the contents of the personal messages that participants wrote along with the petition to see if they matched the goal of the petition. They typically did. Many participants reiterated the petition title (e.g., “be clear”). Some participants expressed a desire for themselves and others to be able to make conscious consumer decisions (e.g., “so that people consciously purchase certain products”), referred to the importance of protecting forests (e.g., “stop using palm oil, because by doing so you demolish the earth's lungs!”), requested the company to stop using palm oil or consider alternatives (e.g., “… can the peanut butter get as tasty without it?”), appealed to justice and moral concerns (e.g., “when you look at your children, you want a nice future for them, right”), or mentioned the necessity of collective effort (e.g., “I would like us to protect the planet all together, meaning that you do your part as well”). Some participants used humor or sarcasm (e.g., “by the way, your sauces are tasty”).

### Confirmatory analyses

**FIGURE 1:**
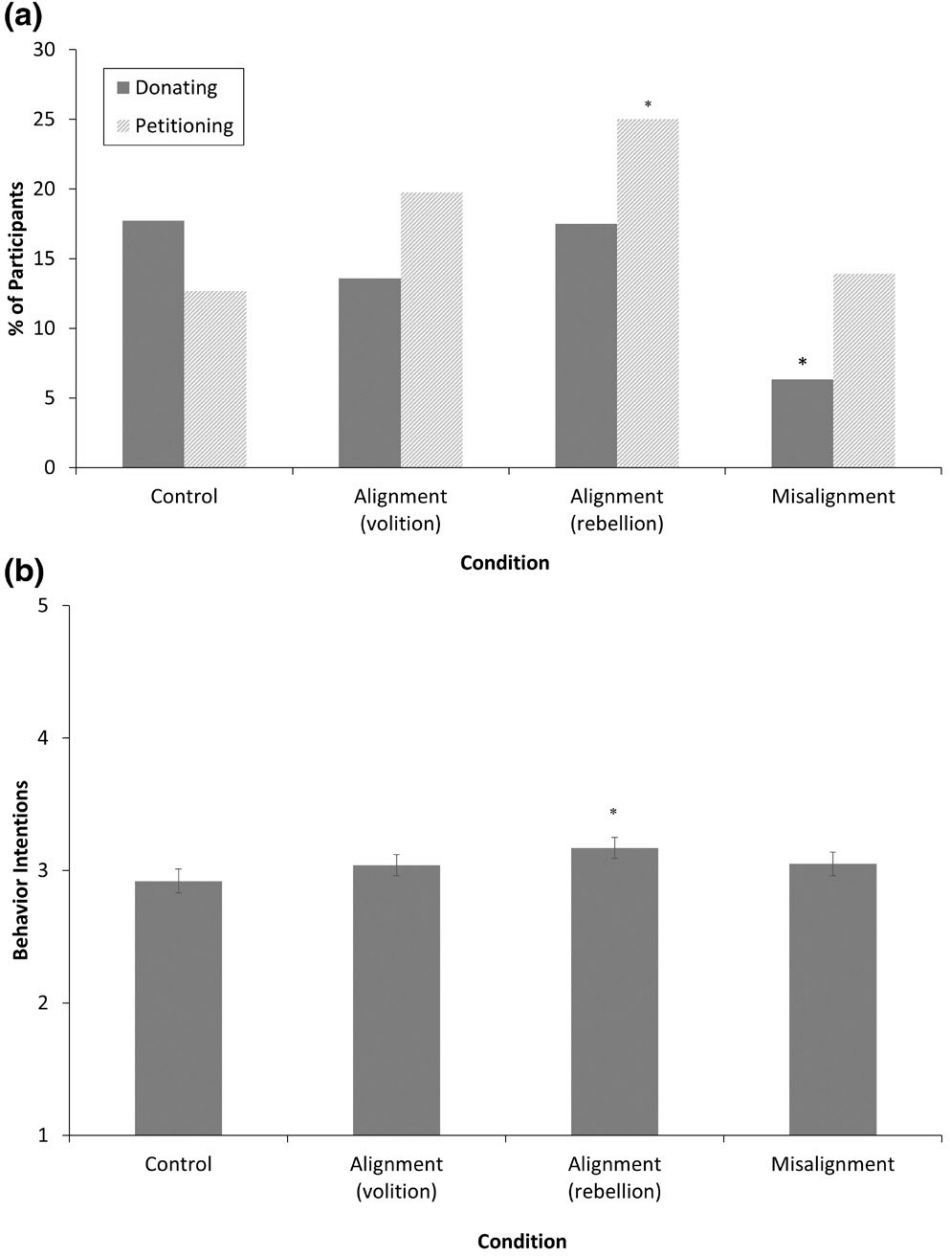
(a) Differences Between Conditions for Pro‐Environmental Donating and Petitioning. (b) Differences Between Conditions for Pro‐Environmental Behavior Intentions. Error bars represent ±1 standard error. *Different from control at *p* < .05.

Figure 1 show the confirmatory contrast tests.

#### Contrasting autonomy‐alignment with control

Participants in the rebellion‐alignment condition were not more likely to donate to environmental charity than those in the control condition, *B* = −0.02 (*SE* = 0.42), Wald(1) = 0.001, *p* = .971, *OR* = 0.99 (95% CI [0.44, 2.23]). Participants in the rebellion‐alignment condition were, however, more likely to sign the petition (25.0% did, compared to 12.7% in the control condition), *B* = 0.83 (*SE* = 0.43), Wald(1) = 3.83, *p* = .050, *OR* = 2.30 (95% CI [0.99, 5.30]), and they reported higher levels of pro‐environmental intentions (*M* = 3.17, compared to *M* = 2.92 in the control condition), *B* = 0.25 (*SE* = 0.12), *F* (315, 1) = 4.52, *p* = .034, *η*_*p*_^*2*^ = .014, *d* = 0.34 (95% CI [0.03, 0.66]).

These effects of enhanced pro‐environmental engagement were specific to rebellion‐alignment and did not generalize to volition‐alignment. Participants in the volition‐alignment condition were not more likely than those in the control condition to donate to environmental charity, *B* = −0.32 (*SE* = 0.44), Wald(1) = 0.52, *p* = .472, *OR* = 0.73 (95% CI [0.31, 1.72]), to sign the petition, *B* = 0.53 (*SE* = 0.44), Wald(1) = 1.46, *p* = .227, *OR* = 1.67 (95% CI [0.72, 4.01]), or to report pro‐environmental intentions, *B* = 0.12 (*SE* = 0.12), *F*(315, 1) = 1.06, *p* = .304, *η*_*p*_^*2*^ = .003, *d* = 0.16 (95% CI [−0.15, 0.47]).

Together, these findings provide partial support for our first hypothesis: autonomy‐alignment increases adolescents' pro‐environmental intentions and petitioning (but not donating) behavior if it appeals to rebellion, but not if it appeals to volition.

#### Contrasting prescriptive misalignment with control

Participants in the prescriptive misalignment condition were less likely to donate to environmental charity than participants in the control condition (6.3% and 17.7% did, respectively), *B* = −1.16 (*SE* = 0.55), Wald(1) = 4.48, *p* = .034, *OR* = 0.31 (95% CI [0.11, 0.92]). Participants in the prescriptive misalignment condition were, however, not less likely to sign the petition, *B* = 0.11 (*SE* = 0.47), Wald(1) = 0.06, *p* = .815, *OR* = 1.12 (95% CI [0.45, 2.80]), nor did they report lower levels of pro‐environmental intentions, *B* = 0.13 (*SE* = 0.12), *F*(315, 1) = 1.22, *p* = .270, *η*_*p*_^*2*^ = .004, *d* = 0.17 (95% CI [−0.14, 0.48]). Again, these findings partially support our hypothesis: autonomy‐misalignment decreases adolescents' engagement in pro‐environmental donating (but not petitioning or intended) behavior.

### Robustness analyses

Confirmatory findings were largely robust to excluding participants who indicated that they encountered video malfunctions (*N* = 8), did not complete the experiment individually (*N* = 7), or failed the attention check (*N* = 9). Only the effects of rebellion‐alignment on petitioning and behavioral intentions were no longer significant (*p* = .051 and .070, respectively) when we excluded participants who failed the attention check (Supplemental Material, Table S2). Furthermore, all confirmatory findings were robust to controlling for relevant demographic variables (i.e., age, educational level), and for whether participants completed the study in the classroom or online (Supplemental Material, Table S3,S4). We also conducted non‐preregistered item‐level analyses for pro‐environmental intentions, which suggest that the effects of rebellion‐alignment on participants' pro‐environmental intentions may be primarily driven by the item capturing nonactivist public sphere behavior (i.e., “I am going to tell people around me how important it is that there are no trees being cut down in the tropical rainforest”; see Supplemental Material for details).

We observed fairly wide confidence intervals for our confirmatory tests, possibly due to the fact that, across conditions, only a minority of participants donated or petitioned. Therefore, we corroborated our confirmatory tests with an alternative statistical approach. We chose a Bayesian approach, which is suited to deal with small numbers of observations (Hoijtink et al., 2019; van de Schoot et al., 2017). The results from these non‐preregistered Bayesian analyses replicated our confirmatory effects, attesting to the robustness of our findings (see Supplemental Material for details).

### Exploratory analyses

When we directly contrasted the rebellion‐alignment and volition‐alignment conditions, we found no significant differences in terms of adolescents' likelihood to donate, *B* = 0.30 (*SE* = 0.44), Wald(1) = 0.47, *p* = .493, *OR* = 0.74 (95% CI [0.32, 1.75]), to sign the petition, *B* = 0.30 (*SE* = 0.38), Wald(1) = 0.64, *p* = .425, *OR* = 0.74 (95% CI [0.35, 1.56]), or to report pro‐environmental behavior intentions, *B* = 0.13 (*SE* = 0.12), *F*(315, 1) = 1.22, *p* = .270, *η*_*p*_^*2*^ = .004, *d* = 0.18 (95% CI [−0.13, 0.49]). Thus, while our confirmatory analyses showed that rebellion alignment, but not volition alignment, motivated pro‐environmental petitioning and intentions, our exploratory analyses that directly contrasted both autonomy‐alignment conditions found no significant differences between them.

### Specificity analyses

We also conducted non‐preregistered analyses pertaining to donating in general, rather than to environmental charities only, which allowed us to study the specificity of the effects of donating. We repeated all donation analyses, but replaced the environmental donation outcome measure by a general donation measure (i.e., aggregating all donations). Importantly, these analyses demonstrated that all significant donation effects were specific to environmental donations, and did not generalize to all donations (see Supplemental Material).

## DISCUSSION

The current preregistered randomized experiment tested an autonomy motive‐alignment approach to promoting adolescents' pro‐environmental behavior. Participants viewed a campaign‐style video that explained the causes of climate change (all conditions), and additionally framed pro‐environmental behavior as a personal choice (volition‐alignment condition), opportunity to rebel (rebellion‐alignment condition), or mandatory (prescriptive misalignment condition). As predicted, we found that rebellion‐alignment increased adolescents' pro‐environmental intentions and the likelihood that they signed a pro‐environmental petition (but not the likelihood that they donated money to environmental charity). Furthermore, prescriptive misalignment decreased the likelihood that adolescents donated to environmental charity (but not the likelihood that they signed a pro‐environmental petition or endorsed pro‐environmental intentions). Different from what we predicted, volition‐alignment did not increase pro‐environmental intentions or behavior.

### Theoretical implications

These findings build on an emerging body of evidence suggesting that adolescents' developmentally salient motives can be harnessed to promote behavior change (Thomaes et al., 2023; Yeager et al., 2018). We applied this motive‐alignment rationale, for the first time, to the domain of pro‐environmental behavior. In doing so, we showed that motive‐alignment is not only effective to promote behaviors that benefit the self (e.g., healthy eating, limiting social media use;Bryan et al., 2016, 2019; Galla et al., 2021), but also behaviors that transcend self‐interest and benefit a collective cause. Motive‐alignment can foster adolescents' motivation to contribute to societal goals (Crone & Fuligni, 2019; Fuligni, 2019).

Our research also extends prior work in that we tested the effects of aligning behavior with adolescents' autonomy motive, specifically, rather than with multiple motives (e.g., autonomy and status motives) simultaneously (Bryan et al., 2016, 2019; Galla et al., 2021). More specifically, we demonstrated that autonomy motive‐alignment increased pro‐environmental engagement when it appealed to adolescents' inclination to rebel (i.e., their drive to take a stand against injustice, or to stand up for noble causes), but not when it appealed to adolescents' need for volition (their drive to freely and willingly make personal choices).

What may account for these findings? We suggest that, along with adolescents' enhanced desire for autonomy, moral developmental advances (e.g., increased concern for social justice; Crone, 2013; Krettenauer & Victor, 2017) encourage adolescents to stand up to authorities that limit freedom or obstruct fairness and justice (e.g., deceptive, manipulative companies; Bryan et al., 2016; Crone, 2013; van Petegem et al., 2015). Appealing to adolescents' tendency to be “moral rebels” (Sonnentag & Barnett, 2016) may be especially powerful given that the climate crisis is intertwined with issues of intergenerational and geographical inequity. For example, the generations and world regions that are most vulnerable to the climate crisis are not the ones that caused it (Sanson & Bellemo, 2021; United Nations International Children's Emergency Fund [UNICEF], 2021). While not all adolescents are prone to overtly rebel, appeals to challenge injustice also fit well with related adolescent characteristics, such as their increased propensity to engage in risk taking (Blakemore & Mills, 2014; Crone & Dahl, 2012). Challenging authorities in the context of climate change can be seen as a form of positive risk taking—while certain means for doing so are contentious, holding authorities to account for a common good generally reflects healthy youth development and civic engagement (Duell & Steinberg, 2019, 2021). Future research could aim to disentangle the exact psychological process that drives adolescents' pro‐environmental engagement following communication that appeals to their rebellious tendencies.

At first blush, our finding that volition‐alignment did not increase pro‐environmental outcomes may seem at odds with prior work that successfully appealed to adolescents' volition to promote their effort and achievement in various domains (Vansteenkiste et al., 2005; Vansteenkiste, Simons, Lens, et al., 2004; Vansteenkiste, Simons, Soenens, & Lens, 2004). It is possible, though, that appealing to volition is most effective in learning contexts that are not just autonomy‐supportive but also provide structure and guidance. Here, guidance could involve, for example, helping adolescents explore how they could support climate change mitigation in accordance with who they are and what they value (Aelterman et al., 2019; Vansteenkiste et al., 2012). Indeed, the importance of providing adolescents with guidance to support their autonomous decisions is increasingly acknowledged in sustainability education (Borg et al., 2012; Olsson et al., 2022).

Furthermore, we found that motive‐(mis)alignment influenced the assessed outcomes in different ways. While appealing to adolescents' rebellious tendency (i.e., rebellion‐alignment) promoted their pro‐environmental behavior intentions and motivated them to sign a petition, it did not motivate them to donate to environmental charity. Moreover, while framing pro‐environmental behavior as an unavoidable duty (i.e., prescriptive‐misalignment) demotivated adolescents to donate, it did not decrease their pro‐environmental behavior intentions or deter them from signing the petition. One explanation for the discrepant behavioral findings may be that petitioning particularly appeals to adolescents' tendency to rebel, more so than donating, because of its directness. Petitioning allows adolescents to directly speak up for themselves and personally contribute to reshaping societal injustice, while donating allows them to do so only indirectly, via charities that do this for them. A complementary explanation may be that donating to environmental charity is relatively costly (Kaiser & Lange, 2021)—more so than petitioning or exhibiting pro‐environmental intentions, which does not cost money and is relatively effortless (Boyes & Stanisstreet, 2012)—and may thus require more persuasive power.

This raises the question of whether promoting low‐cost pro‐environmental behavior is meaningful in the first place. There is reason to assume that it is. First, low‐cost pro‐environmental behaviors can be impactful when adopted by many (e.g., reusing shopping bags; Kaiser & Lange, 2021; Nielsen et al., 2021). Second, low‐cost pro‐environmental behaviors can be important as “gateway” behaviors: for some individuals, the initial enactment of low‐cost pro‐environmental behavior paves the way for the subsequent enactment of more costly forms of pro‐environmental behavior (Truelove et al., 2014). Such positive spillover can occur via increased perceptions of efficacy and environmental identity (Geiger et al., 2021; Lauren et al., 2016; van der Werff et al., 2014). Adolescence may be a particularly sensitive period for such effects—as a time of rapid growth and foundational learning, initial behavior changes that originate in adolescence can “snowball” into longer‐term trajectories of pro‐environmental engagement (Crone & Dahl, 2012; Dahl et al., 2018).

### Practical implications

Current policies that aim to engage adolescents with the climate crisis primarily focus on raising awareness and fostering knowledge of climate change and its solutions (Borg et al., 2012). While such education‐based policies tend to exert positive effects, their typical emphasis on long‐term impacts and prescription of “desired” or “required” behaviors is not optimally tailored to adolescents' sensitivity to short‐term rewards and need for autonomy (Crone & Dahl, 2012; Rodríguez‐Meirinhos et al., 2020; Yeager et al., 2018). The current research provides, for the first time, a proof‐of‐concept for how such educational policies may be improved: by incorporating strategies that speak to adolescents' autonomy motive. By casting pro‐environmental behavior as an opportunity to rebel against unjust authorities, pro‐environmental behavior becomes more rewarding in the here and now (Bryan et al., 2016; Thomaes et al., 2023). The appeal of taking a stand against unjust authorities may also be one explanation for why climate protests, such as the Friday for Future marches, have gained traction among young people: many adolescents see these protests as a means to show that they are old enough to have a say and to oppose the injustices that they see (de Moor, De Vydt, et al., 2020; IPCC, 2022; Jorgenson et al., 2019).

### Limitations and future directions

Our study also has limitations. First, it was designed to test a proof‐of‐concept for promoting in‐the‐moment pro‐environmental behavior in a controlled setting. Future work could explore the implementation of motive‐alignment techniques in policies implemented in real‐world contexts with the goal of promoting longer‐term pro‐environmental behavior change. The relatively modest effects that we found underscore that motive‐alignment techniques should be used to complement rather than substitute traditional policies. Second, while we measured different types of pro‐environmental behavior, we cannot know to what extent our findings generalize to other pro‐environmental behaviors. Future work could test such generalizability to public sphere (e.g., volunteering, protesting) or private‐sphere (e.g., adopting a plant‐based diet) behaviors (Nielsen et al., 2021). Third, while we focused on adolescents specifically, autonomy motive‐alignment may be effective to motivate pro‐environmental engagement in individuals of other ages as well—that is, indeed, autonomy is a salient motive across the life‐course (Deci & Ryan, 1985, 2000)—a possibility that could be explored in future work. Finally, although the need for autonomy is likely universal, its manifestation may differ between cultures (Kagitcibasi, 2005). For example, autonomy may be less likely to manifest in rebellion in countries that are traditionally more authority‐loyal as compared to the Netherlands, where we conducted our study. Cross‐cultural work is needed to test generalizability and potentially explore cultural adaptations to optimize the motivating power of autonomy‐alignment.

## CONCLUSION

Adolescents are driven to contribute to society, and taking action on climate change is an opportune way to do so. Yet, despite adolescents' widespread concern about climate change, their typical engagement in pro‐environmental behavior lags behind. Our research illustrates a novel approach to promoting adolescents' pro‐environmental behavior: by aligning the behavior with their autonomy motive, and especially their desire to stand up to unjust authorities. Motive alignment has the potential to help adolescents contribute to a sustainable future for themselves and generations to come.

## CONFLICT OF INTEREST STATEMENT

None.

## Supporting information


Data S1.

## Data Availability

The data that support the findings of this study are available on request from the corresponding author. The data are not publicly available due to privacy or ethical restrictions.
