# Supplementary material for: Promoting adolescents' pro‐environmental behavior: A motive‐alignment approach
Source: J Res Adolesc. 2024 Dec 10;35(1):e13044. doi: 10.1111/jora.13044 (PMC11758481; doi:10.1111/jora.13044)
Supplement: Supplementary file 1 — Data S1. [file JORA-35-0-s001.docx]

**Supplemental Material**

**Measure of Autonomy Motive-Alignment**

We assessed autonomy motive-alignment with pro-environmental behavior using four items from the recently validated Sustainability Motive Alignment Scale (Grapsas et al., 2023), which we adapted to match the content of our experimental manipulations (“By donating money to environmental organizations, you can express what you value as a person”; “When I support environmental organizations, I do so because I want to [and not because somebody tells me to]”; “By calling out on companies’ behavior, you can stand up to the pollution that these companies cause”; “Young people like me are fed up with large companies doing little to protect the tropical rainforest”; 1 = *totally disagree* to 5 = *totally agree*; *M* = 3.65; *SD* = .59). Although we initially planned to explore whether the effects of the experimental manipulation on pro-environmental outcomes were mediated by autonomy motive-alignment, we dropped this analysis because we were unable to measure this construct reliably (Cronbach’s *α* = .56).

**Fidelity Check**

To test the fidelity of our manipulations, we collected online data in a separate sample of 244 Dutch adolescents aged 16-20 (*M_age_* = 18.80, *SD_age_* = 1.11, 68.4% female, 79.1% white) via Pollfish in May and June 2024. As in our main study, participants were randomly selected to watch one of four versions of our video (control *N* = 62; rebellion-alignment *N* = 61; volition-alignment *N* = 59; and prescriptive-misalignment *N* = 62). We assessed manipulation fidelity with 4 items tapping into educational (“The video stated that many trees in tropical rainforests are cut down for palm oil production”), rebellion-aligned (“The video encouraged people to rise against unfair companies”), volition-aligned (“The video showed that people can support environmental charities if that fits with who they are”), and prescriptive-misaligned (“The video stated that people must support environmental charities, as if they cannot make their own choice”) messaging. Participants rated their agreement with each item on a 5-point scale (1 = *totally disagree*, 5 = *totally agree*). Descriptive statistics are reported in Table S1.

We analyzed the data using linear regression with planned contrasts, regressing the items on experimental condition. Compared to participants in the control condition, participants in the rebellion-aligned condition more strongly endorsed that “The video encouraged people to rise against unfair companies”, *B* = 0.62 (*SE* = 0.18), *F*(240, 1) = 11.97, *p* < .001, *η_p_^2^* = .05, *d* = 0.93 (95% CI [0.30, 1.03]); participants in the volition-aligned condition more strongly endorsed that “The video showed that people can support environmental charities if that fits with who they are”, *B* = 1.03 (*SE* = 0.18), *F*(240, 1) = 31.88, *p* < .001, *η_p_^2^* = .17, *d* = 0.99 (95% CI [0.66, 1.42]); and participants in the prescriptive-misaligned condition more strongly endorsed that “The video stated that people must support environmental charities, as if they cannot make their own choice”, *B* = 1.42 (*SE* = 0.20), *F*(240, 1) = 51.75, *p* < .001, *η_p_^2^* = .18, *d* = 1.18 (95% CI [0.82, 1.58]). These findings demonstrate the fidelity of our experimental manipulations, suggesting that the rebellion-alignment, volition-alignment, and prescriptive-misalignment videos were indeed perceived as such.

**Table S1**

*Fidelity Check Descriptives for Each Condition*

|  | *M* (*SD*) | | | |
| --- | --- | --- | --- | --- |
| Item | Rebellion | Volition | Control | Prescriptive |
| The video stated that many trees in tropical rainforests are cut down for palm oil production | 4.20 (0.91) | 4.39 (0.77) | 3.98 (1.02) | 4.39 (0.82) |
| The video encouraged people to rise against unfair companies | 4.18 (0.89) | 2.98 (1.11) | 3.56 (0.97) | 3.63 (0.98) |
| The video showed that people can support environmental charities if that fits with who they are | 3.38 (0.80) | 3.90 (1.03) | 2.87 (0.95) | 2.52 (1.18) |
| The video stated that people must support environmental charities, as if they cannot make their own choice | 2.10 (0.91) | 1.97 (1.10) | 2.53 (1.13) | 3.95 (1.23) |

**Robustness Analyses**

Table S2 presents the results of robustness analyses excluding participants that (a) encountered video malfunctions, (b) indicated they did not complete the experiment individually, or (c) failed the attention check in the experimental session. Table S3 presents correlations between study variables. Table S4 presents the results of robustness analyses controlling for relevant demographic variables and for whether participants completed the study in the classroom or online. Table S5 presents the results of non-preregistered item-level analyses for pro-environmental intentions.

Furthermore, to address the relatively wide confidence intervals for our confirmatory findings, we corroborated them with a different statistical approach. We chose a Bayesian approach, which is suited to deal with small numbers of observations (van de Schoot et al., 2017). Specifically, we used Bayesian informative hypothesis testing to evaluate the relative support for our preregistered hypotheses: (1) rebellion-alignment > control, (2) volition-alignment > control, (3) prescriptive misalignment < control. We did so for each of the pro-environmental outcomes (i.e., donating, petitioning, behavior intentions). We evaluated the relative support for our hypotheses using Bayes Factors (BF). BFs reflect the extent to which the data support our hypothesis compared to all other possible hypotheses. A BF of 1 indicates equal support for our hypothesis and all possible alternatives; a BF > 1 indicates support in favor of our hypothesis (Hoijtink et al., 2019). For example, a BF of 10 would indicate that our hypothesis is 10 times more likely than all other possible hypotheses. We conducted our Bayesian analyses using the bain package in R (Gu et al., 2021; R Core Team, 2022). We used the default, non-informative priors.

Our Bayesian analyses replicated the preregistered confirmatory analyses that we report in the manuscript (see Table S6): the BFs supported our hypothesis that, compared to participants in the control condition, participants in the rebellion-alignment condition were more likely to sign the petition (BF = 46.82) and reported higher levels of pro-environmental intentions (BF = 58.63). The BFs also supported the hypothesis that participants in the prescriptive misalignment condition were less likely to donate to environmental charity (BF = 9.72). While our preregistered confirmatory analyses suggested that the effects of rebellion-alignment on petitioning and pro-environmental intentions did not generalize to volition-alignment, the BFs suggest that they might (BFs = 7.31 and 5.61, respectively). We chose not to interpret these findings, though, as they are not consistent across statistical methods, and the strength of the evidence (i.e., magnitude of the BFs) is relatively small.

**Specificity Analyses**

Specificity analyses showed that our confirmatory donation findings were specific to pro-environmental donations (i.e., donations to environmental charities). Participants were not more likely to donate to any charity (i.e., aggregated across environmental and nonenvironmental donations) in the volition-alignment condition, *B* = –0.16 (*SE* = 0.35), Wald(1) = 0.20, *p* = .653, *OR* = 0.86 (95% CI [0.43, 1.70]), and in the rebellion-alignment condition, *B* = –0.08 (*SE* = 0.35), Wald(1) = 0.05, *p* = .822, *OR* = 0.93 (95% CI [0.47, 1.83]), relative to the control condition. Similarly, participants were not less likely to donate to any charity in the prescriptive condition, *B* = –0.19 (*SE* = 0.35), Wald(1) = 0.28, *p* = .597, *OR* = 0.83 (95% CI [0.42, 1.67]), relative to the control condition. Finally, we did not find that participants in the volition- and rebellion-alignment conditions differed from each other in how much they donated to any charity, *B* = –0.08 (*SE* = 0.35), Wald(1) = 0.05, *p* = .822, *OR* = 0.92 (95% CI [0.46, 1.84]). These findings thus attest to the specificity of our main findings.

**Table S2**

*Test Statistics of Robustness Checks for Preregistered Confirmatory Tests*

|  |  | Test statistics | | |
| --- | --- | --- | --- | --- |
| Robustness check excluding | Contrast | Donating | Petitioning | Behavioral intentions |
| 1. Video malfunctions (*N* = 8) | Volition vs. control | *B* = –0.27 (*SE* = 0.44), Wald(1) = 0.38, *p* = .536, *OR* = 0.76 (95% CI [0.32, 1.80]) | *B* = 0.58 (*SE* = 0.44), Wald(1) = 1.73, *p* = .188, *OR* = 1.78 (95% CI [0.75, 4.23]) | *B* = 0.14 (*SE* = 0.12), *F*(1, 307) = 1.31, *p* = .253, *η_p_^2^* = .004 |
|  | Rebellion vs. control | *B* = 0.00 (*SE* = 0.42), Wald(1) = 0.00, *p* = 1.00, *OR* = 1.00 (95% CI [0.44, 2.27]) | *B* = 0.85 (*SE* = 0.43), Wald(1) = 3.99, *p* = .046, *OR* = 2.35 (95% CI [1.02, 5.41]) | *B* = 0.27 (*SE* = 0.12), *F*(1, 307) = 4.91, *p* = .027, *η_p_^2^* = .016 |
|  | Prescriptive vs. control | *B* = –1.16 (*SE* = 0.55), Wald(1) = 4.48, *p* = .034, *OR* = 0.31 (95% CI [0.11, 0.92]) | *B* = 0.11 (*SE* = 0.47), Wald(1) = 0.06, *p* = .815, *OR* = 1.12 (95% CI [0.45, 2.80]) | *B* = 0.14 (*SE* = 0.12), *F*(1, 307) *=* 1.39, *p* = .239, *η_p_^2^* = .005 |
| 2. Not completed individually (*N* = 7) | Volition vs. control | *B* = –0.30 (*SE* = 0.44), Wald(1) = 0.47, *p* = .492, *OR* = 0.74 (95% CI [0.31, 1.75]) | *B* = 0.55 (*SE* = 0.44), Wald(1) = 1.55, *p* = .214, *OR* = 1.73 (95% CI [0.73, 4.09]) | *B* = 0.13 (*SE* = 0.12), *F*(1, 308) = 1.23, *p* = .269, *η_p_^2^* = .004 |
|  | Rebellion vs. control | *B* = –0.90 (*SE* = 0.42), Wald(1) = 0.05, *p* = .832, *OR* = 0.91 (95% CI [0.34, 2.10]) | *B* = 0.85 (*SE* = 0.43), Wald(1) = 3.99, *p* = .046, *OR* = 2.35 (95% CI [1.02, 5.41]) | *B* = 0.26 (*SE* = 0.12), *F*(1, 308) = 4.76, *p* = .030, *η_p_^2^* = .015 |
|  | Prescriptive vs. control | *B* = –1.38 (*SE* = 0.59), Wald(1) = 5.46, *p* = .019, *OR* = 0.25 (95% CI [0.08, 0.80]) | *B* = 0.13 (*SE* = 0.47), Wald(1) = 0.07, *p* = .790, *OR* = 1.13 (95% CI [0.45, 2.85]) | *B* = 0.12 (*SE* = 0.12), *F*(1, 308) = 1.00, *p* = .317, *η_p_^2^* = .003 |
| 3. Failed attention check (*N* = 9) | Volition vs. control | *B* = –0.43 (*SE* = 0.45), Wald(1) = 0.90, *p* = .342, *OR* = 0.65 (95% CI [0.27, 1.57]) | *B* = 0.45 (*SE* = 0.44), Wald(1) = 1.03, *p* = .310, *OR* = 1.57 (95% CI [0.66, 3.74]) | *B* = 0.10 (*SE* = 0.12), *F*(1, 306) = 0.67, *p* = .413, *η_p_^2^* = .002 |
|  | Rebellion vs. control | *B* = –0.04 (*SE* = 0.42), Wald(1) = 0.01, *p* = .920, *OR* = 0.96 (95% CI [0.42, 2.20]) | ***B* = 0.84 (*SE* = 0.43), Wald(1) = 3.77, *p* = .052, *OR* = 2.31 (95% CI [0.99, 5.36])** | ***B* = 0.22 (*SE* = 0.12), *F*(1, 306) = 3.32, *p* = .070, *η_p_^2^* = .011** |
|  | Prescriptive vs. control | *B* = –1.15 (*SE* = 0.55), Wald(1) = 4.38, *p* = .036, *OR* = 0.32 (95% CI [0.11, 0.93]) | *B* = 0.13 (*SE* = 0.47), Wald(1) = 0.07, *p* = .790, *OR* = 1.13 (95% CI [0.45, 2.85]) | *B* = 0.13 (*SE* = 0.12), *F*(1, 306) = 1.08, *p* = .299, *η_p_^2^* = .004 |

*Note.* Robustness tests in bold diverge from initial findings.

**Table S3**

*Correlations Between Demographics, Location of Participation, and Pro-Environmental Outcomes*

| Variable | *N* | Donating | Petitioning | Behavioral intentions |
| --- | --- | --- | --- | --- |
| Age (in years) | 315 | .19* | –.03 | –.02 |
| Girl (vs. boy) | 310 | .02 | –.05 | .08 |
| Lower educational track (vs. higher educational track) | 315 | .15* | –.02 | –.12* |
| Middle educational track (vs. higher educational track) | 315 | –.22* | –.18* | –.20* |
| Online participation (vs. in classroom participation) | 319 | .20* | .09 | .14* |

* *p* < .05.

**Table S4**

*Test Statistics of Robustness Checks for Preregistered Confirmatory Tests*

| Pro-environmental outcome | Robustness check controlling for | Contrast | Test statistics |
| --- | --- | --- | --- |
| Donating | Age, educational track, online participation | Volition vs. control | *B* = –0.12 (*SE* = 0.48), Wald(1) = 0.06, *p* = .815, *OR* = 0.89 (95% CI [0.35, 2.30]) |
|  |  | Rebellion vs. control | *B* = 0.08 (*SE* = 0.47), Wald(1) = 0.03, *p* = .860, *OR* = 1.09 (95% CI [0.43, 2.72]) |
|  |  | Prescriptive vs. control | *B* = –1.15 (*SE* = 0.56), Wald(1) = 3.83, *p* = .050, *OR* = 0.32 (95% CI [0.10, 1.00]) |
| Petitioning | Educational track | Volition vs. control | *B* = 0.72 (*SE* = 0.46), Wald(1) = 2.44, *p* = .118, *OR* = 2.05 (95% CI [ 0.83, 5.04]) |
|  |  | Rebellion vs. control | *B* = 1.05 (*SE* = 0.45), Wald(1) = 5.52, *p* = .019, *OR* = 2.87 (95% CI [ 1.19, 6.90]) |
|  |  | Prescriptive vs. control | *B* = 0.27 (*SE* = 0.49), Wald(1) = 0.31, *p* = .577, *OR* = 1.31 (95% CI [0.50, 3.42]) |
| Behavioral intentions | Educational track, online participation | Volition vs. control | *B* = 0.13 (*SE* = 0.12), *F*(1, 308) = 1.29, *p* = .257, *η_p_^2^* = .004 |
|  |  | Rebellion vs. control | *B* = 0.26 (*SE* = 0.12), *F*(1, 308) = 5.14, *p* = .024, *η_p_^2^* = .016 |
|  |  | Prescriptive vs. control | *B* = 0.16 (*SE* = 0.12), *F*(1, 308) = 1.87, *p* = .172, *η_p_^2^* = .006 |

**Table S5**

*Test Statistics of Item-Level Analyses for Pro-Environmental Intentions*

| Pro-Environmental Intentions Item | Contrast | Test statistics |
| --- | --- | --- |
| 1. I plan to join actions to help protect the forests | Volition vs. control | *B* = -0.03 (*SE* = 0.14), *F*(1, 315) = 0.04, *p* = .845, *η_p_^2^* < .001 |
|  | Rebellion vs. control | *B* = 0.04 (*SE* = 0.14), *F*(1, 315) = 0.06, *p* = .802, *η_p_^2^* < .001 |
|  | Prescriptive vs. control | *B* = -0.06 (*SE* = 0.14), *F*(1, 315) = 0.20, *p* = .659, *η_p_^2^* = .001 |
| 2. I am going to tell people around me how important it is that there are no trees being cut down in the tropical rainforest | Volition vs. control | *B* = 0.23 (*SE* = 0.16), *F*(1, 315) = 2.23, *p* = .136, *η_p_^2^* = .007 |
|  | Rebellion vs. control | *B* = 0.48 (*SE* = 0.16), *F*(1, 315) = 9.39, *p* = .002, *η_p_^2^* = .029 |
|  | Prescriptive vs. control | *B* = 0.15 (*SE* = 0.16), *F*(1, 315) = 0.93, *p* = .335, *η_p_^2^* = .003 |
| 3. I want to try to eat or use less products that contain palm oil, because trees may have been cut down for those products | Volition vs. control | *B* = 0.16 (*SE* = 0.15), *F*(1, 315) = 1.07, *p* = .302, *η_p_^2^* = .003 |
|  | Rebellion vs. control | *B* = 0.24 (*SE* = 0.15), *F*(1, 315) = 2.38, *p* = .124, *η_p_^2^* = .007 |
|  | Prescriptive vs. control | *B* = 0.30 (*SE* = 0.15), *F*(1, 315) = 3.91, *p* = .049, *η_p_^2^* = .012 |

**Table S6**

*Bayesian Informative Hypothesis Tests*

| Pro-environmental outcome | Hypothesis test | BF |
| --- | --- | --- |
| Donating | Rebellion > control | 0.77 |
|  | Volition > control | 0.35 |
|  | Prescriptive < control | 9.72 |
| Petitioning | Rebellion > control | 46.82 |
|  | Volition > control | **7.31** |
|  | Prescriptive < control | 0.72 |
| Behavioral intentions | Rebellion > control | 58.63 |
|  | Volition > control | **5.61** |
|  | Prescriptive < control | 0.16 |

*Note.* BF = Bayes Factor, a value > 1 indicates support for our hypothesis. BFs in bold deviate from our preregistered confirmatory analyses.
